# Enterobacterial LPS-inducible LINC00152 is regulated by histone lactylation and promotes cancer cells invasion and migration

**DOI:** 10.3389/fcimb.2022.913815

**Published:** 2022-07-25

**Authors:** Jianwei Wang, Zhi Liu, Yuyu Xu, Yipeng Wang, Fei Wang, Qingqing Zhang, Chunhua Ni, Yi Zhen, Rui Xu, Qisha Liu, Weijia Fang, Ping Huang, Xingyin Liu

**Affiliations:** ^1^ Department of Pathogen Biology-Microbiology Division, Globe of health center, Nanjing Medical University, Nanjing, China; ^2^ Key Laboratory of Pathogen of Jiangsu Province and Key Laboratory of Human Functional Genomics of Jiangsu Province, Nanjing Medical University, Nanjing, China; ^3^ Institute of Pediatric Research, Children’s Hospital of Soochow University, Suzhou, China; ^4^ Department of Surgery , the Third Affiliated Hospital, Nanjing Medical University, Nanjing, China; ^5^ Cancer Biotherapy Center, First Affiliated Hospital, School of Medicine, Zhejiang University, Hangzhou, China; ^6^ Key Laboratory of Holistic Integrative Enterology, The Second Affiliated Hospital of Nanjing Medical University, Nanjing, China

**Keywords:** lipopolysaccharide, lncRNA, histone lactylation, colorectal cancer, enteric bacteria

## Abstract

Gut microbes participate in pathogenesis by interacting with the host genome through epigenetic mechanisms, such as long non-coding RNAs. However, the mechanisms by which the microbiota induce expression alteration of long non-coding RNAs remains unclear. Here, we quantified the transcriptome alteration of human colon cell lines after being infected by a common enteric pathogen *Salmonella typhimurium SL1344*. We observed a widespread lncRNAs expression alteration. Among them, the elevated expression of LINC00152 was verified and proved to be induced by enteric bacteria-derived lipopolysaccharide (LPS). The inducible LINC00152 were found to inhibit *Salmonella* invasion and inflammation response. LINC00152 was overexpressed in tumors of the clinical CRC samples compared with adjacent normal tissues. Accordingly, we also demonstrated that overexpression of LINC00152 promoted the migration and invasion of colorectal cancer cells. Consistently, we observed an increased abundance of gram-negative bacteria and LPS in tumors tissue. Taken together, the above data implicated that enriched gram-negative bacteria in tumor tissue might promote tumor growth through modulating the expression of LINC00152. Furthermore, we demonstrated that LPS upregulated the expression of LINC00152 by introducing histone lactylation on its promoter and decreasing the binding efficiency of the repressor, YY1, to it. Our results provide new insights into how enterobacteria affect host epigenetics in human disease.

## Introduction

Gut microbes are involved in the pathogenesis of multiple human diseases, including obesity, inflammatory bowel disease, metabolic disease, and cancers (Ananthakrishnan et al., 2017; Dai et al., 2018; Ponziani et al., 2019; Zhong et al., 2019). For example, *Salmonella* is a zoonotic pathogen which is rising global concern to human and animal health (Knodler and Elfenbein, 2019). Its infection in humans can be chronic and increase the risk of inflammatory bowel diseases (IBD), gallbladder carcinoma, and colorectal cancer (CRC) (Gradel et al., 2009; Kato et al., 2013; Scanu et al., 2015). Studies have linked dysbiosis of the gut microbiome and tumor-associated bacteria to colorectal cancer (CRC) (Xia et al., 2020). Fusobacterium nucleatum, one of the most prevalent bacterial species in colorectal cancer tissues, has been repeatedly reported to promote colorectal neoplasms (Zackular et al., 2013; Mima et al., 2016; Yu et al., 2017; Rubinstein et al., 2019).

Accumulating evidence has shown that long non-coding RNAs (lncRNAs) are involved in multiple aspects of human health and diseases. For example, in colorectal cancer, lncRNA FAL1 promotes tumorigenesis by regulation of miR-637/NUPR1 pathway (Wang et al., 2019). The lncRNA MALAT1 is involved in the progression or metastasis in multiple tumors, including lung cancer (Gutschner et al., 2013), breast cancer (Kim et al., 2018), and gastric adenocarcinoma (Lu et al., 2019). HULC accelerates liver cancer (Xin et al., 2018) and promotes the proliferation, migration, and invasion of HCC cells *in vitro* and *in vivo* (Zhang et al., 2019b). LINC00152 (ENSG00000222041, CYTOR) promotes colorectal cancer metastasis by interacting with β-catenin (Yue et al., 2018).

Multiple studies have demonstrated the roles of lncRNAs in pathogenic infection. For example, the transcriptional profile of lncRNAs is dynamically altered upon pathogenic infection, suggesting that lncRNAs are involved in the host immune response or propagation of pathogens (Shirahama et al., 2020). Additionally, stimulating human primary monocytes with lipopolysaccharide (LPS), the main cell wall component of gram-negative bacteria, induces lncRNAs expression and then regulates host immune response (Ilott et al., 2014). However, the mechanism by which the pathogens induce transcript alteration of long non-coding RNAs remains unclear. Recently, In CRC/colorectal adenomas, Xia et al. discovered novel associations between abundance of tumor-associated bacteria and ubiquitous hypermethylation of tumor suppressor genes promoters (Xia et al., 2020), which implicated the tumor-associated bacteria-derived metabolites play intriguing roles in regulating gene expression related to cancer development through epigenetics machinery. Nevertheless, evidence supporting direct interactions between gut microbiota and non-coding RNAs in CRC development remains limited.

In the present study, to determine whether enteric pathogenic bacteria regulate the expression of lncRNAs and the associated role in colon cancer development, we firstly quantified the transcriptome alteration of human colon cell lines after being infected by enteric pathogenic, *Salmonella typhimurium* (*S.typhimurium*) *SL1344* to investigate the lncRNA alterations upon the infection. We discovered that LINC00152, an oncogenic lncRNA, dramatically increased in HCT116 cells when infected with *S.typhimurium SL1344* or treated with other enteric pathogenic bacteria-derived LPS. Moreover, its overexpression inhibited inflammation in response to bacterial cells and increased migration and invasion of CRC cells. We further revealed that LPS regulates LINC00152 expression by introducing histone lactylation, a new epigenetic modification (Chiariotti et al., 2016) to activate its transcription.

## Materials and methods

### Cell culture

Human colonic epithelial HCT116 (RRID: CVCL_0291) and HEK293T (RRID: CVCL_0063) were purchased from the American Type Culture Collection (ATCC, Manassas, Virginia). The mouse macrophage RAW 264.7 cells (RRID: CVCL_0493) was from Yunzhi Chen Lab. Normal epithelial cell line FHC is from Xiaoqing Yuan Lab (RRID: CVCL3688). All cell lines used in this study have been authenticated using STR profiling within the last three years. All cells were cultivated in Dulbecco’s modified eagle medium (DMEM) supplemented (Thermo, 11965092, USA) with 10% fetal bovine serum (FBS, Thermo, 10099141, USA) and penicillin-streptomycin at 37°C, as previously described (Liu et al., 2011). All experiments were performed with mycoplasma-free cells.

### Bacterial strains and growth condition

The *E. coli 0157:H7* strain was routinely cultured overnight (37°C, 200 rpm) in LB until to an optical density (OD 600 nm) of 0.6 to have a final density to 5× 10^8^ ± 1 × 10^8^ CFU/mL. The *F. nucleatum* strain was purchased from ATCC (*F nucleatum* subsp. nucleatum ATCC 25586) and was cultured as described previously (Yang et al., 2017). In brief, *F. nucleatum* was grown in Columbia blood agar supplemented (Haibo, HBPM0153, China) with 5 µg/ml haemin, 5% defibrinated sheep blood (5%), and 1 µg/ml vitamin K1 in an anaerobic glove box at 37°C. For inactivation, bacteria (10^9^ cfu/ml) were washed with sterile PBS for three times and heat-killed at 100°C for 20 min., and then stored at 80°C as described previously (Ruggeri et al., 2015). The *S.typhimurium* SL1344 strains used in this study is from Jun Sun Lab. The nonagitated cultures were prepared by inoculating monoclonal bacteria into 5 ml of Luria-Bertani broth for 6 hrs, then transferring 0.05 ml of the stationary phase culture into 50 ml medium followed by incubation at 37°C for 18 hrs, as previously described (Lu et al., 2012).

### RNA extraction, cDNA library preparation

HCT116 cells were infected with *S*.*typhimurium*-containing HBSS (1.6×10^10^ bacteria/ml) or HBSS for 30 minutes, washed 3 times in HBSS and incubated at 37°C for 6 hours. Total RNA was extracted from *S*.*typhimurium*-infected and control cells with HBSS treatment using TRIzol reagent (Invitrogen, USA). A total of 3μg RNA per sample was used for sample preparations. Firstly, ribosomal RNA was removed by Epicentre Ribo-zero™ rRNA Removal Kit (Epicentre, RZNB1056, USA). Subsequently, the rRNA-depleted RNA was used for libraries construction with NEBNext^®^ Ultra™ Directional RNA Library Prep Kit (NEB, USA). Finally, products were purified (AMPure XP system) and library quality was assessed by the Agilent Bioanalyzer 2100.

### RNA sequencing and processing

Raw data were quality controlled by fastp to remove low-quality reads. The resulting clean reads were aligned to the reference genome (hg38) using TopHat v2.0.9 (Kim et al., 2013). The sequencing quality statistics are listed in **Table S1**. The transcripts of each sample were assembled by Cufflinks (v2.1.1) (Trapnell et al., 2012). Cuffdiff software was used to calculate FPKMs (Fragment Per Kilobase of exon model per Million mapped reads) of both lncRNAs and coding transcripts in each sample. Differential genes were calculated by the Student t-test based on the log-transformed FPKM value. Genes with FDR < 0.05 and fold change >=2 were considered statistically different. The raw data have been deposited into CNGB Sequence Archive (CNSA) (Guo et al., 2020) of China National GeneBank DataBase (CNGBdb) with accession number CNP0001877.

### 16S rRNA sequence process

The 16S rRNA sequencing data for colon tumors and adjacent tissues were downloaded from SRA with accession SRP104334 (Gao et al., 2017). The sequencing reads were quality controlled with fastp software (Chen et al., 2018), then processed using QIIME2 pipeline to obtain the abundance of bacteria at the genus level (v2019.10) (Bolyen et al., 2019, 2). Differential genera were identified using paired Wilcoxon signed-rank test based on the relative abundance, and those with a *P*-value less than 0.05 were defined as significant. The functional profile of the gut microbial community was predicted using PICRUSt2 (Douglas et al., 2020, 2). Predicted functional genes were categorized into KEGG Orthology (KO). Statistical differences in KO frequencies were determined with the paired Wilcoxon signed-rank test with FDR <0.05 and fold change >=1.5.

### Histopathological analysis for the tumor and adjacent tissues

A cross-section of the patients with colon cancer was embedded in paraffin, sectioned, and stained with Hematoxylin and Eosin (H&E) after being fixed in paraformaldehyde per previous method (Lu et al., 2017).

### Immunofluorescence

Immunofluorescence staining was performed as previously described (Wang et al., 2018a). Briefly, the tumor and adjacent tissues were freshly isolated, fixation with 10% neutral buffered formalin, and then embedded in paraffin wax. Sections of the paraffin-embedded human colon tissues were used for immunostaining. First, slides were placed in 60°C ovens for 20 minutes, then 2–4 μm thick sections were incubated in two 50 mL washes of xylene for 10 minutes each time. Sections were then incubated successively in 50 mL washes of 100%, 95%, and 75% ethanol for 5 minutes each. Slides were washed in 50 mL PBS for 5 minutes twice. The epitope was retrieved by boiled 0.01M citric acid buffer (PH6.0) for 10-15 minutes, then cooled slides on the benchtop to room temperature. Specimen was blocked in 100 μL 5% goat serum blocking buffer for 30 min to reduce nonspecific background. The permeabilized tissue samples were incubated with anti-LPS (1:200, Cell Signaling, 14011S, USA) dilutions for 10 to 12 hours at 4°C. Specimens were then incubated with DAPI Stain Solution (YEASEN, 40728ES03, China) for 10 minutes at room temperature and then coated cover slip. Specimens were examined with a FluoView FV1200 laser scanning confocal microscope (Olympus).

### Establishment of HCT116 cells stably expressing LINC00152

The lentiviral expression vector pLenti-EF1-Puro-LINC00152 was constructed by inserting a XbaI-NotI fragment containing the human LINC00152 complementary DNA (cDNA) into the pLenti-EF1-Puro plasmid (7.095 kb). PCR primers for generating a XbaI-NotI fragment containing the LINC00152 cDNA were as follows: XbaI-LINC00152 forward primer: 5-TCTAGAACTGACAAAACTACCGAACC-3; LINC00152-NotI backward primer: 5-GCGGCCGCGTTTTCTTTAGTTTTGCTT-3. After sequencing, purified plasmids were transfected into 293T cells together with packaging plasmids, psPAX2 and pMD2G, using polyjet reagent with ratio 4:3:1. The supernatants were collected after 48 h for virus purification. The purified virus was transfected into HCT116 cells. After 48 h, cells were passaged and cultured using medium containing puromycin (60210ES25, YEASEN, China) at a final concentration of 0.5 uM to obtain HCT116 cell line with stably expressing LINC00152. The cell line was used to perform bacterial infection experiment, q-PCR assay, and invasion and migration assay for cancer cells.

### *S. typhimurium* attachment and invasion of human epithelial monolayers

HCT116 cells with stably overexpressing of LINC00152 were infected with *S. typhimurium* according to the previously described method (Liu et al., 2011). After infection, the cell-associated bacteria and the internalized bacteria were assessed per previous method (Liu et al., 2012). In short, the cell-attached bacteria are released by the incubation of 100 ul of 1% Triton X-100 (Sigma). The bacteria internalized in epithelial cells were released with 1% Triton X-100 after gentamicin treatment for 20 min. A 0.9 ml sample of LB medium was added and mixed vigorously with both the cell-attached and the internalized samples, respectively, and then use MacConkey agar to determine their CFUs.

### Real-time quantitative PCR analysis

Total RNA was extracted from epithelial cells treated with LPS (Biosharp, BS904, China), lactic acid (L1750, Sigma, Germany), or heat-killed *S. typhimurium/E. coli 0157:H7*/*Fusobacterium nucleatum* and normal/proximal colonic tumor tissue using TRIzol reagent. Gel electrophoresis was used to verify RNA integrity. RNA was reverse transcribed using the HiScript^®^ II Q Select RT SuperMix for qPCR (+gDNA wiper) (Vazyme, R233-01, China) according to the manufacturer’s protocol. The RT cDNA reaction products were subjected to quantitative real-time PCR using the Hieff^®^ qPCR SYBR Green Master Mix (YEASEN, 11202ES03, China). The primers used for qPCR were shown in **Table S2**.

### Assay level of lactic acid

Lactate concentrations in supernatants of cells cultured for 24 hours were measured by a lactate acid assay kit (Solarbio Science & Technology, China) according to the manufacturer’s suggestions.

### Western blot

Cell line cultures of infected or control cells were rinsed twice in ice-cold PBS and lysed in protein loading buffer (50 mM Tris, pH 6.8, 100 mM dithiothreitol, 2% SDS, 0.1% bromophenol blue, and 10% glycerol). Equal amounts of protein were separated by SDS-PAGE, transferred to nitrocellulose membranes, and analyzed by immunoblotting with anti-Histone 4 (PTM BIOLABS, PTM-1009, China), anti-H4K5lac (PTM BIOLABS, PTM-1407, China), anti-H4K8lac (PTM BIOLABS, PTM-1415, China), anti-YY1 (Proteintech, 22156-1-AP, China), and anti-β-actin antibodies (Bioworld, BS6007M, China). After probing the membranes with an infrared-conjugated secondary antibody, the signals were visualized and quantitated using LI-COR Odyssey v3.0 software.

### Chromatin immunoprecipitation

HCT116 cells were cross-linked with 1% fresh formaldehyde for 15 min at room temperature, neutralized with glycine for 5 min and lysed in lysis buffer. The cross-linked DNA was then sheared into fragments ~200-1000 bp in length with Biosafer. Sheared chromatin was immunoprecipitated with anti-H4K8lac, anti-H3K18lac, anti-H4K5lac, anti-H3K27ac, and anti-YY1 antibodies using protein A/G agarose (Pierce, 20421, USA). Mouse IgG (Cell Signaling Technology, 5946, USA) was used as a mock antibody for negative control. Finally, the immunoprecipitated DNA was de-crosslinked and isolated. ChIP-qPCR were performed with the Hieff^®^ qPCR SYBR Green Master Mix(Vazyme, R233-01, China). Antibody binding signals were calculated as a percentage of input chromatin precipitated for each region examined. ChIP-qPCR primers (**Table S3**) were designed for the LINC00152 promoter based on the Histone H3K27Ac and H3K4me3 binding site (Zhang et al., 2019a).

### Patient population and clinical data

Thirteen pairs of CRC tissues and adjacent normal tissues were collected from patients diagnosed with CRC at the Third Affiliated Hospital, Nanjing Medical University (Nanjing, China). Tumor and tumor adjacent normal tissues were obtained during surgical treatment at the Department of General Surgery. Adjacent normal tissues were taken about 2-3 cm proximal to the tumor. The samples were isolated and snap-frozen in liquid nitrogen immediately. All samples were stored at − 80°C before use. All patients were given written informed consent to participate in the study. The study was approved by the research ethics committee at the Third Affiliated Hospital, Nanjing Medical University (2018-SR-24).

### Cancer cell migration and invasion assay

Cells growing in the log phase were trypsinized, resuspended in a serum-free medium, and seeded into chambers (8-μm pore size in a polycarbonate membrane) (Corning, CLS3422, USA). The chambers were coated with Matrigel (BD Biosciences, 356234, USA) for cell invasion assays. Pre-thawed Matrigel was diluted 1:8 using serum-free medium, pipet to mix well, then add 100 ul diluted Matrigel in the chamber. The plates were left to incubate for 2 h at 37°C for gelling. Cells were washed with PBS, detached with trypsin, and counted with a Neubauer hemocytometer. The cell suspension concentration was adjusted to 1x10^5^ cells/mL and 100 ul of cell suspension was added to each well and spread evenly by means of soft shaking. Medium with 20% FBS (750 μl) was added to the lower chamber. After incubation for 72 hours, the cells on the top surface of the insert were removed with a cotton swab. Cells that had migrated to the bottom surface of the insert were stained in 0.1% crystal violet for 30 minutes, rinsed in PBS, and subjected to microscopic inspection. Images of four random fields (10×) were captured from each membrane, and the number of migratory or invasive cells was counted. The migration and invasion results were normalized to cell number under the same treatment conditions. Triplicate assays were performed for each experiment.

### Statistics

Unless otherwise indicated, data are presented as mean ± SEM from at least three independent biological replicates. The student’s t-test was used to analyze differences between two groups of samples. The one-way ANOVA was performed to analyze differences among three or more groups. All statistical tests were two-sided, and *P*-values less than 0.05 indicated statistical significance.

## Results

### Bacteria-derived LPS regulate long non-coding RNA expression

**Figure 1:**
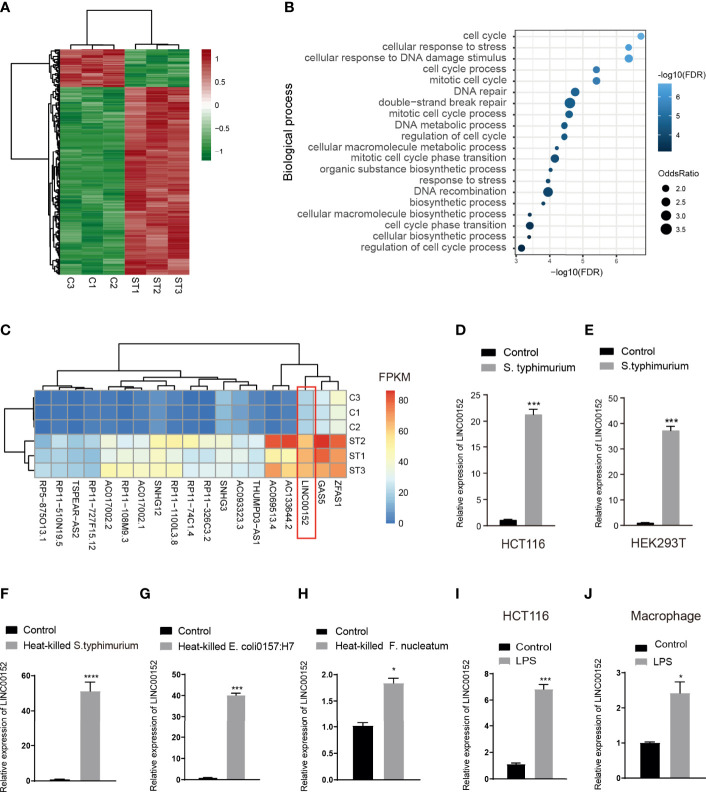
LPS induced alteration of lncRNA expression. **(A)** Heatmap of lncRNA expression in *Salmonella* infected human intestinal epithelial HCT116 cells (ST) and controls(C). **(B)** The enriched biological process of differential lncRNAs based on the co-expressed protein-coding genes. **(C)** The heatmap of the top 20 expressed lncRNA in the control and *Salmonella* infected HCT116 cells. **(D, E)** qPCR validation of the elevated expression of LNC_ LINC00152 in the *S. typhimurium* infected HCT116 cells **(D)** and HEK293T cells **(E)**. **(F, H)** The expression of LINC00152 in the heat-treated gram-negative bacteria-infected HCT116. **(I, J)** The expression of LINC00152 in the LPS treated HCT116 and macrophage. *P < 0.05; ***P < 0.001 ****P < 0.0001. All experiments were in three replicates.

To understand how pathogens reshape transcription profiles of the long non-coding RNAs, we infected human intestinal epithelial HCT116 cells with wild-type *S.typhimurium* strain SL1344. Cells were incubated with SL1344 or HBSS for 6 hours, and then the RNA was extracted for RNA sequencing. We identified 187 differentially expressed lncRNAs (DELs) in *S.typhimurium* treatment cells (FDR<0.05, |log2 (fold change)|>1) (**Figure 1A**, **Table S4**). Co-expression analyses between the DELs and protein-coding genes indicated that the altered lncRNAs might be associated with the biological process of cell cycle and DNA repair (**Figure 1B**). **Figure 1C** showed the top 20 expressed lncRNAs among the DELs, including GAS5, SNHG12, SHNG3, and LINC00152. LINC00152 is proposed as a key oncogenic lncRNA in human cancers, and the elevated LINC00152 expression was significantly associated with poor prognosis in multiple cancer types (Liang et al., 2018). To validate the RNA sequencing results, we performed a qRT-PCR assay after incubating HCT116 or a human embryonic kidney cell HEK293T with live *S.typhimurium*. Consistently, LINC00152 was significantly upregulated in the *S.typhimurium*-treated HCT116 and HEK293T cells (**Figures 1D, E**).

Lipopolysaccharide (LPS) is a natural adjuvant synthesized by Gram-negative bacteria involved in the onset and progression of inflammation and metabolic diseases. To test whether the LPS attributes the microbiota-induced transcriptional alteration, we treated HCT116 cells with three heat-treated gram-negative bacteria, i.e., *S. typhimurium, E.coli 0157:H7*, and *Fusobacterium nucleatum (F. nucleatum)*. LINC00152 was significantly upregulated in the treated cells (**Figures 1F-H**). We then treated the HCT116 and the mouse macrophage RAW 264.7 cells with LPS. Similarly, the expression of LINC00152 was increased in the LPS-treated HCT116 and macrophage cells (**Figures 1I, J**).

### LINC00152 inhibited bacteria-induced inflammation and promoted colon cancer cell invasion and migration

**Figure 2:**
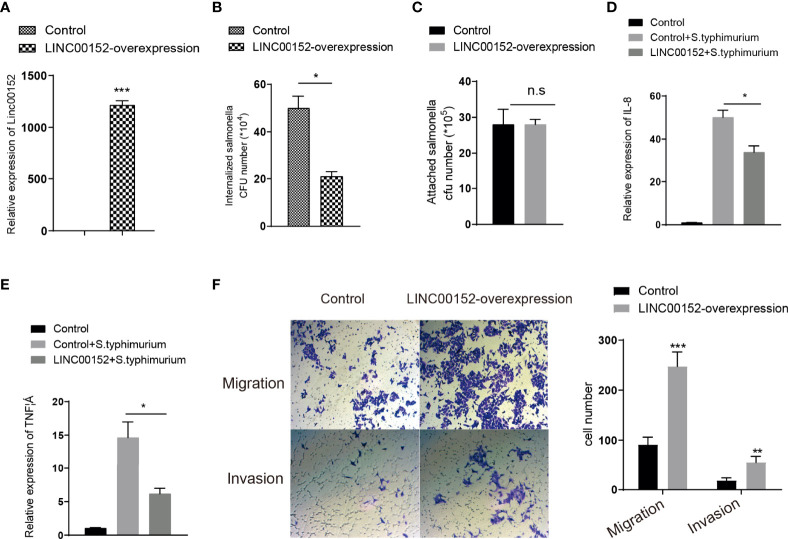
LINC00152 is involved in the *S. typhimurium-*introduced inflammation and cancer cell invasion and migration. **(A)** The expression level of LINC00152 in the control and overexpressed stalely cells. **(B)** LINC00152 overexpressed cells had fewer internalized *Salmonella*. **(C)** The number of bacteria attachment with cells in control and LINC00152-overexpressed cells**. (D, E)** The expression level of IL-8 and TNF α were reduced in the LINC00152 overexpressed cells compared with control HCT116 cells infected with *S. typhimurium*. **(F)** The migration and invasion of LINC00152 overexpressed HCT116 cells. All experiments were in three replicates. *P < 0.05; **P < 0.01; ***P < 0.001; n.s: not significant.

Previous studies have shown that *S. typhimurium* colonization increases the inflammatory response (Zhang et al., 2018). To explore whether *Salmonella*-induced upregulation of LINC00152 is responsible for host responses such as inhibition of bacterial infection, the number of *Salmonella* that invaded human intestinal epithelial HCT116 cells with normal or overexpressed LINC00152 levels (**Figure 2A**) was counted. As compared to control cells with normal LINC00152 expression, cells with overexpressing LINC00152 reduced the number of internalized *Salmonella* SL1344 bacteria into the epithelial cells (**Figure 2B**). In contrast, the number of bacteria associated with cells did not differ significantly between the two groups (**Figure 2C**). To further determine the role of LINC00152 in *Salmonella*-epithelial cell interactions, we hypothesized that the overexpressed LINC00152 contributes to the inhibition of inflammatory responses. Consistent with our prediction, we found that cells with overexpressed LINC00152 downregulated the level of the inflammatory cytokine IL-8 and TNF-a induced by *Salmonella* colonization (**Figures 2D, E**).These results indicated that the elevated LINC00152 expression inhibited the *S. typhimurium-*induced inflammation through possibly lowering the number of internalized bacteria into the host cells.

Overexpression of LINC00152 was observed in colon cancer (Yue et al., 2016) and breast cancer (Shen et al., 2019). Next, to detect the role of LINC00152 in colon cancer development, we assess whether overexpression of LINC00152 promotes the cancer cell migration and invasion. We found that the abilities of migration and invasion in the LINC00152-overexpressed cells were dramatically increased compared with control cells (**Figure 2F**). Taken together, these data suggested that the enterobacterial LPS-induced upregulation of LINC00152 might promote the development of CRC *via* changing tumor microenvironment.

### Altered lncRNA expression and bacteria abundance in colon cancer tissue

**Figure 3:**
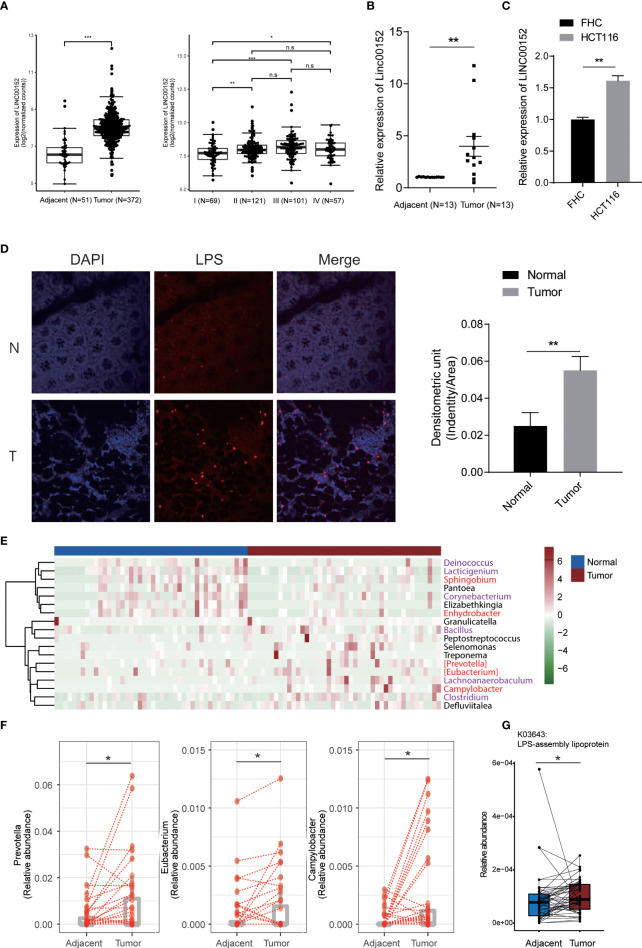
lncRNA expression and bacteria abundance of CRC samples. **(A-C)** The expression of LINC00152 in TCGA colorectal samples **(A)**, our clinical CRC samples **(B)**, and the HCT116 cell **(C)**. **(D)** The level of LPS in tumor tissue and the adjacent normal mucosa. Experiment was performed in three replicates. **(E)** The differential species between tumor tissues (N=44) and the adjacent normal ones (N=44). Red and purple labeled species are gram-positive and negative bacteria, respectively. **(F)** The expression of gram-negative bacteria in tissue and normal samples. **(G)** The increased LPS-assembly lipoprotein in tumor tissue compared with normal tissue adjacent to the tumor. *P < 0.05; **P < 0.01; ***P < 0.001; n.s: not significant.

We then examined the LINC00152 in the colorectal tumor tissue in human clinical samples. The analysis of RNA sequencing data from CRC cohort in TCGA database revealed that a dramatical upregulation of LINC00152 expression in the tumor tissues compared with the adjacent normal tissues (**Figure 3A**). In addition, the expression of LINC00152 increased with the development of tumor stage, especially from stage I to stage II (**Figure 3A**). We also recruited 13 CRC patients and quantified the expression of LINC00152 in tumor and tumor-adjacent tissues. The representative HE staining images for tumor tissue and the adjacent normal mucosa were shown in **Figure S1**. Similarly, higher LINC00152 expression was observed in tumor tissues (**Figure 3B**) and CRC tumor cell line (**Figure 3C**). We then detected the level of LPS in these clinical samples. A notably higher level of LPS in the tumor tissues was observed compared with the adjacent normal mucosa (**Figure 3D**).

Multiple studies have reported significant differences in the microbiome composition in colon tumor tissue related to normal tissue (Castellarin et al., 2012; Dejea et al., 2014; Burns et al., 2015). Therefore, we examined the 16S rRNA sequencing data of the CRC tumor and the adjacent normal tissues. We observed enrichment of multiple gram-negative pathogenic bacteria in the tumor tissues compared with the adjacent normal ones (**Figure 3E**). For example, *Prevotella*, *Eubacterium*, and *Campylobacter* (**Figure 3F**). *Prevotella* species are anaerobic Gram-negative bacteria of the Bacteroidetes phylum with increased abundance in CRC mucosa and inflammatory disorders (Flemer et al., 2017; Larsen, 2017). PICRUSt2 (Douglas et al., 2020, 2) was used to generate the differential functional profile of gut microbiota between two groups. We found 1280 KOs (KEGG Orthology) differed substantially in tumor in comparison to those in tissue adjacent to tumor (**Table S5**). Impressively, consistent with differential genus analysis for gram-negative bacteria (**Figure 3F**), increased LPS-assembly lipoprotein in tumor tissue indicated elevated levels of LPS compared with normal tissue adjacent to the tumor (**Figure 3G**).

Taken together, the result implicated that the increased gram-negative bacteria in tumor tissue might be responsible for the LPS-induced lncRNA alteration.

### LPS upregulates LINC00152 through histone lactylation

**Figure 4:**
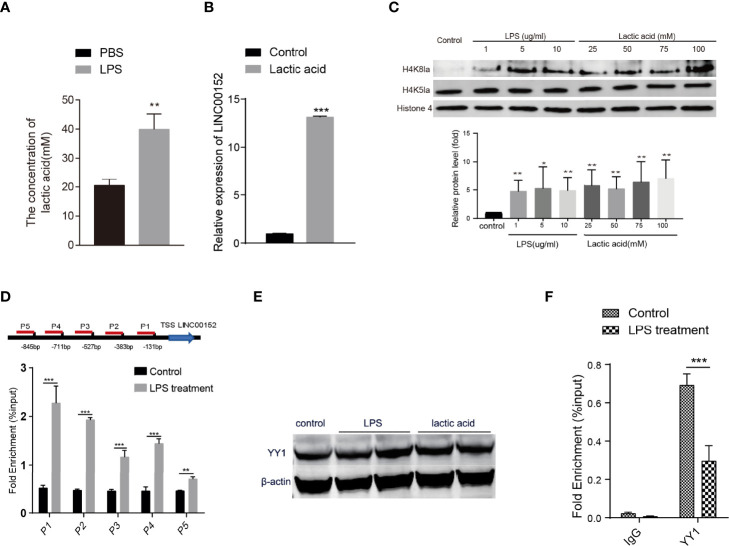
The epigenetic modifications of bacteria-introduced lncRNA. **(A)** LPS induced an elevated level of lactic acid in HCT116 cells. **(B)** The expression of LINC00152 level in the lactic acid-treated cells (N=3). **(C)** The level of histone lysine lactylation in the LPS treated cells (N=3). **(D)** ChIP-qPCR revealed that the binding signaling of H4K8la to the LINC00152 promoter regions was higher than in the LPS treated cells compared with control group (N=3). **(E)** The expression of YY1 upon LPS and lactic acid treatment (N=2). **(F)** LPS reduced the binding efficiency of YY1 to the LINC00152 promoter compared with the control group (N=3). *P < 0.05; **P < 0.01 ***P < 0.001.

Studies have reported that LPS could increase lactate levels in the mouse model and human cell lines (Sommer et al., 2018; Zhang et al., 2019a). Lactate-derived histone lysine lactylation is a new epigenetic modification that stimulates gene transcription from chromatin (Zhang et al., 2019a). Based on the previous studies, we hypothesize that the enterobacterial LPS might increase the lactic acid level and induce histone lysine lactylation thus altering the expression of LINC00152. Firstly, we assessed the level of lactic acid in the LPS-stimulated HCT116 cells and observed an elevated level of lactic acid after the LPS stimulation (**Figure 4A**). We then observed significantly increased expression of LINC00152 in the lactic acid-treated HCT116 cells (**Figure 4B**).

To investigate whether LPS could increase the histone Kla levels, we monitored H4K8la, H4K5la, and H3K18la modification in the LPS-treated cells. As shown in **Figure 4C** and **Figure S2A**, the H4K8la level are increased dramatically in LPS and lactic acid-treated cells. In contrast, the level of H4K5la and H3K18la were not affected. We then performed ChIP-qPCR to check whether the promoter regions of LINC00152 have an elevated binding of histone Kla using anti-H4K8la, anti-H4K5la, and anti-H3K18la antibodies. As shown in **Figure 4D**, CHIPqPCR with anti-H4K8la showed signi ficant higher enrichment in the LINC00152 promoter regions in the LPS treated cells compared with the control cells. Nevertheless, no significant differential enrichment of H4K5la and H3K18la were observed in the promoter region of LINC00152 between two groups, respectively (**Figure S2B**). These results suggest that histone lysine lactylation might be a novel mechanism underlying LPS induced gene expression changes.

Shen et al. reported that transcription factor YY1 negatively regulates LINC00152 expression (Shen et al., 2019), so we further check whether YY1 expression changes upon LPS or lactic acid treatment. As shown in **Figure 4E**, neither LPS nor lactic acid affected the expression of YY1. However, ChIP-qPCR showed that LPS significantly reduced the binding efficiency of YY1 to the promoter region of LINC00152 compared with the control group (**Figure 4F**). This result implicated that LPS treatment and bacterial infection might upregulate LINC00152 expression by affecting the YY1 binding efficiency.

## Discussion

**Figure 5:**
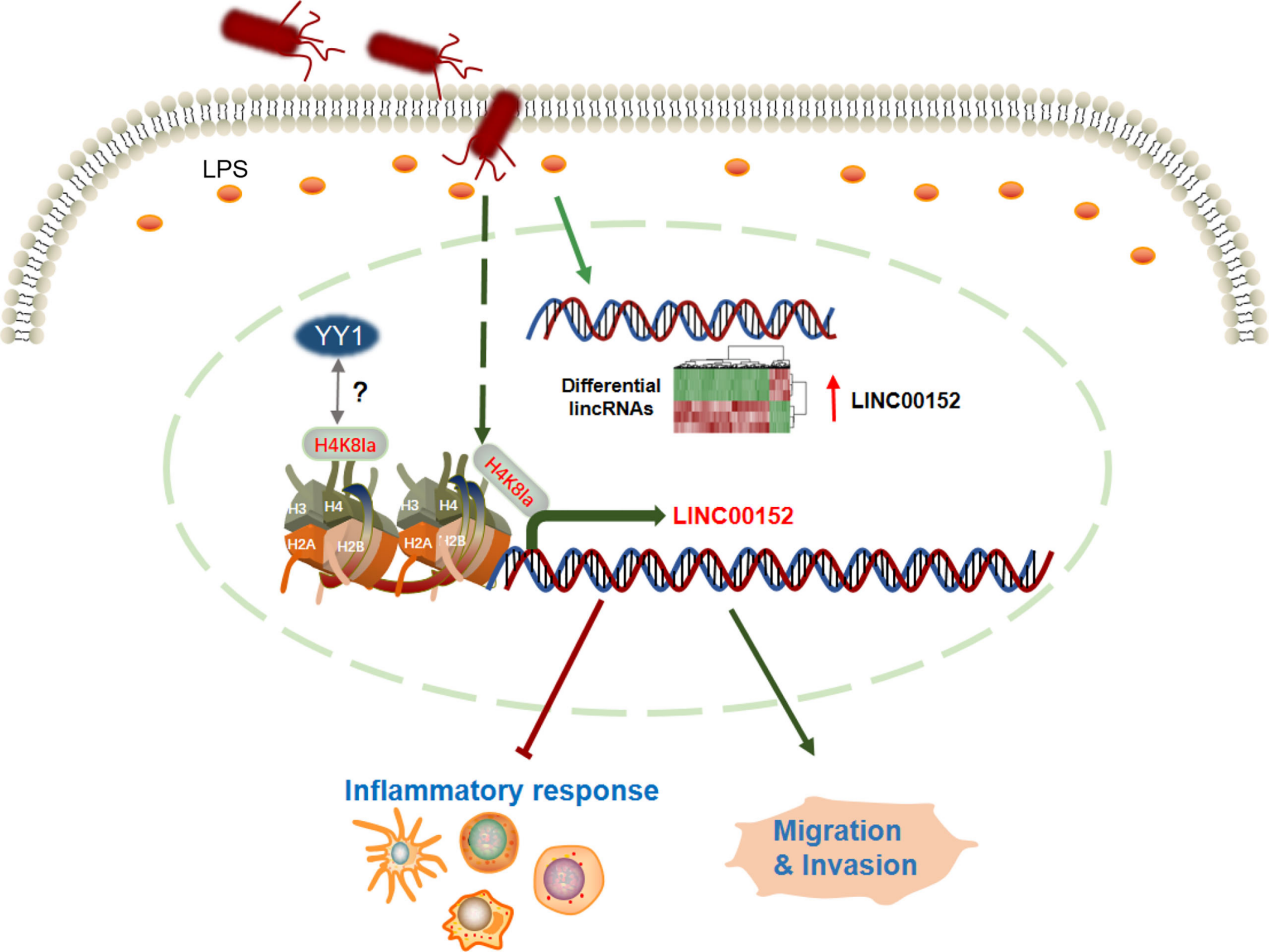
The graphic summary of this study. The enterobacterial LPS induced widespread expression alteration of lncRNAs, including LINC00152. The bacteria-derived LPS upregulates the expression of LINC00152 by introducing histone lactylation on its promoter, and decreasing the binding efficiency of YY1 to it. The LPS-induced overexpression of LINC00152 was linked to the inflammation response and cancer cells migration and invasion.

In our present study, as shown in **Figure 5**, we demonstrated that Enterobacterial *Salmonella* infection altered lncRNA mRNA expression in intestinal epithelial cells. We further provide evidence that enteric bacterial LPS regulates candidate lncRNA, LNC000152 expression linking to intestinal inflammation and migration and invasion of the cancer cells. Additionally, we demonstrated that LPS upregulated the expression of LINC00152 by introducing histone lactylation on its promoter. Moreover, our results revealed that LPS treatment decreased the binding efficiency of YY1, a negative expression regulator, to the LINC00152 promoter.

Microbiome analysis in tumor tissues showed alterations compared with normal tissues (Nejman et al., 2020). Moreover, recent studies indicated that opportunistic pathogenic gut microbes such as *Fusobacterium nucleatum* mediated CRC development and therapy (Mima et al., 2016; Yu et al., 2017). However, the bacterial interaction with the host to involve in cancer progress remained largely unclear. Our study revealed that the abundance of gram-negative bacteria in the cancer tissue was higher than in the adjacent tissue. It indicated that the microecological structure of the tumor tissue might drive the development of CRC progress through inducing the expression of lncRNAs. Microbiota and intestinal epithelial cells consist of a dynamic complex ecosystem. The microbiome-host immunity interaction influences oncogenesis (Kayama et al., 2020). Notably, we also observed that LPS could induce lncRNA expression in macrophages and overexpression of LINC00152 inhibited expression of proinflammtory cytokines. Therefore, this data led us to speculate that bacteria in tumor tissue may regulate lncRNAs expression in immune cells to affect tumor immune environment. Appropriate probiotics or prebiotics can be used as a dietary supplement to modulate the microbial community CRC patients, thereby reducing the concentration of LPS in tumor tissues, which might be benefit patients.

LPS affects diverse sets of epigenetic factors such as DNA methylation, histone acetylation, histone methylation, and chromatin-associated complexes (Chiariotti et al., 2016). But the whole landscape of how LPS triggered these epigenetic alterations is far from being depicted. The accumulated evidence indicated that the recognition of external LPS stimulus triggers the intracellular signaling cascade which might affect the expression of chromatin factors or enzymes for epigenetic modifications, their recruitment to specific gene promoters, or the chromatin structures.

Histone lysine lactylation is a newly characterized epigenetic modification. It uses lactate as a substrate to generate lactyl-CoA for lysine lactylation on histones to regulate gene expression in diverse pathophysiological conditions, including infection and cancer (Zhang et al., 2019a). Our study showed that LPS and lactic acid treatment dramatically increased the histone lysine lactylation level of the LINC00152 promoter. However, further efforts are needed to reveal the underlying mechanisms by which LPS modified histone lysine lactylation level.

LINC00152 is overexpressed in multiple tumors, including breast cancer, pancreatic cancer, and CRC (Wang et al., 2018b; Liu et al., 2020; Zhu et al., 2020). Elevated LINC00152 expression was considered as a risk factor for tumor invasion, metastasis and associated with the poor prognosis in cancer patients (Liang et al., 2018). However, the upstream mechanism of the upregulation of LINC00152 remains poorly understood. Studies have revealed that the expression of LINC00152 is regulated by transcription factors Sp1 (Gao et al., 2021) and YY1 (Shen et al., 2019). In this study, we have demonstrated that the LPS treatment reduced the binding efficiency of YY1 to the LINC00152 promoter. However, further studies are needed to confirm the role of histone lysine lactylation in the decreased affinity between YY1 and LINC00152 promoter.

The previous studies reported that *Salmonella* infection will induce activation of NF-κB pathway (Itoh et al., 2005; Ning et al., 2011; Kobelt et al., 2020; Wang et al., 2021). Based on the previous studies on Wnt protein (Liu et al., 2012), we hypothesize that the upregulation of LINC00152 is the host reaction to defense against *Salmonella* infection and inhibit enteric bacterial-induced inflammation, similar to the role of upregulation of wnt2 and wnt1 in response to *Salmonella* infection. Hence, we examined whether the upregulated expression of LINC00152 contributes to the activity of the host’s defense, such as inhibition of bacterial internalization by affecting various signaling pathways, for example, NF-κB pathway to modulate the inflammatory response. Consistent with our prediction, our data showed overexpression of LINC00152 reduced the expression of IL-8 and TNF-a, which are targets of NF-κB pathway. On the other hand, *Salmonella* infection will inject its’ effectors into host cells through type III secretion system (T3SS) (Knodler and Elfenbein, 2019), hence, overexpression of LINC00152 might interact with some effectors being secreted from bacteria to inhibit bacterial internalization and further modulate host signaling pathways.

Large amounts of microbiota-derived molecules are taken up by the host, and significantly contribute to the host metabolism and host epigenetic machinery (Mischke and Plösch, 2016). Although other group have also identified that LPS regulate lncRNA expression (Du et al., 2017), whether other microbiota-derived metabolites regulate the expression of lncRNAs keep unknown. Increasing studies indicated that CRC is caused by genetic and epigenetic instability, which triggers the transformation of colon epithelial cells into adenocarcinoma cells and remodels the surrounding stromal tumor microenvironment (Amirkhah et al., 2019). However, the upstream of regulators in epigenomics machinery in CRC development remains largely unknown. Therefore, future studies exploring whether other microbiota-derived metabolites in cancer tissue can regulate lncRNA expression are essential to reveal the transformation mechanism of colon epithelial cells into adenocarcinoma cells. In summary, the current study provides novel molecular insights into host-bacteria interactions in infectious diseases and CRC.

## Data Availability Statement

The datasets presented in this study can be found in online repositories. The names of the repository/repositories and accession number(s) can be found in the article/**Supplementary Material**.

## Ethics Statement

The studies involving human participants were reviewed and approved by Moderate and highly differentiated human adenocarcinoma samples were obtained from the Third Affiliated Hospital, Nanjing Medical University, China, in accordance with approval from the institute (Ethic No. 2018-SR-24). The patients/participants provided their written informed consent to participate in this study.

## Author Contributions

XL and PH conceived the study. FW conducted the experiments during the revision. XL designed the study. JWW, QZ, RX, QL, YW, FW, and JWW conducted the experiment and analyzed the data. ZL analyzed the RNA-seq and metagenomics sequencing data. QZ, YX, YZ, and PH collected and analyzed clinical samples. XL and ZL wrote the original manuscript. XL, ZL, and JWW revised the manuscript. All of the authors interpret and confirm all of the data and manuscript. All authors contributed to the article and approved the submitted version.

## Funding

This work was supported by NSFC grant 81871628 to XYL, NSFC grant 31900123 to QL, NSFC grant 81902027, Natural Science Foundation of Jiangsu Province to JWW (BK20171045), Gusu Health Talents program of Soochow city (GSWS2021028) to JWW, and Innovation project of postgraduate training in Jiangsu Province to QZ (KYCX19_1118).

## Conflict of Interest

The authors declare that the research was conducted in the absence of any commercial or financial relationships that could be construed as a potential conflict of interest.

## Publisher’s Note

All claims expressed in this article are solely those of the authors and do not necessarily represent those of their affiliated organizations, or those of the publisher, the editors and the reviewers. Any product that may be evaluated in this article, or claim that may be made by its manufacturer, is not guaranteed or endorsed by the publisher.

## Supplementary Material

Supplementary Figure 1 The representative HE staining image of tumor (A) and tumor-adjacent tissues (B) from clinical CRC human samples.

Supplementary Figure 2 The level of histone lysine lactylation in LPS or lactic acid-treated cells. **(A)** The level of H3K18la in LPS and lactic acid-treated cells compared with the control group. **(B)** The enrichment of H4K5la and H3K18la in the promoter regions of LINC00152 had no significant difference between the LPS treated group and the control group.
