# Supplementary figures and images for: Enterobacterial LPS-inducible LINC00152 is regulated by histone lactylation and promotes cancer cells invasion and migration
Source: Front Cell Infect Microbiol. 2022 Jul 25;12:913815. doi: 10.3389/fcimb.2022.913815 (PMC9359126; doi:10.3389/fcimb.2022.913815)

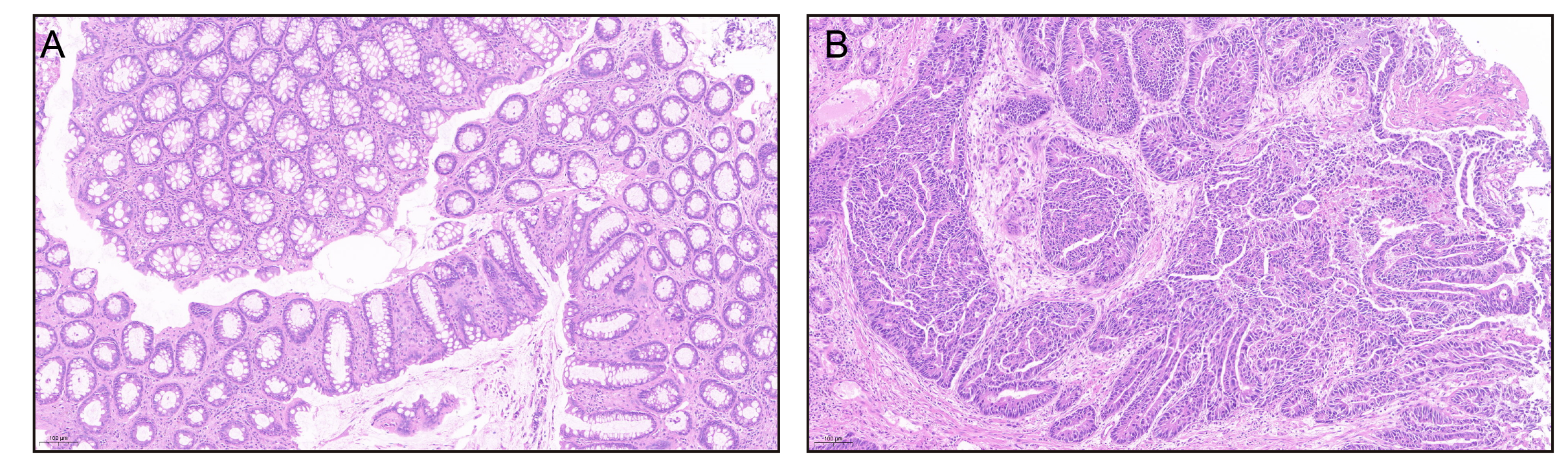

Supplement: Supplementary Figure 1 — The representative HE staining image of tumor (A) and tumor-adjacent tissues (B) from clinical CRC human samples. [file Image_1.tif]

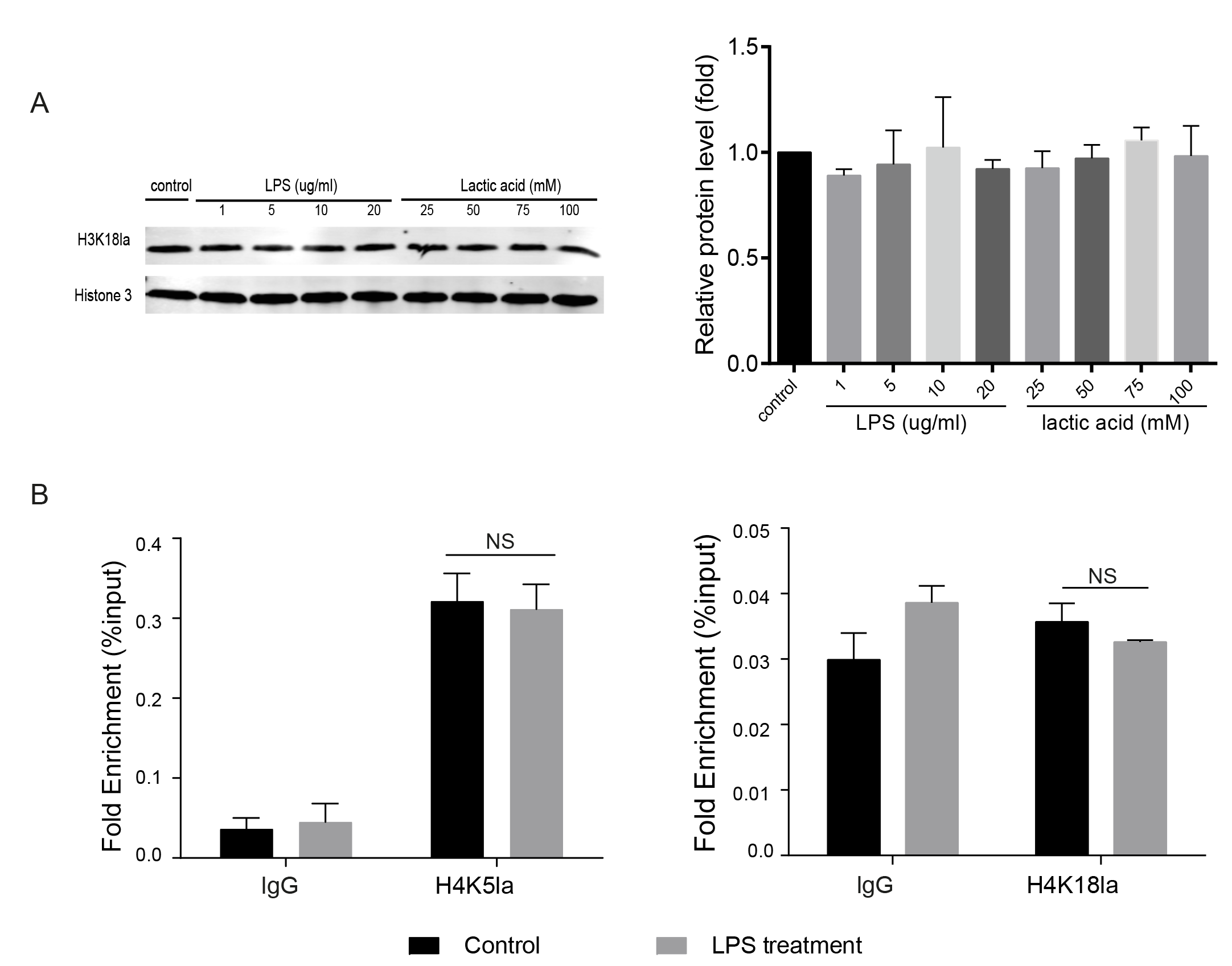

Supplement: Supplementary Figure 2 — The level of histone lysine lactylation in LPS or lactic acid-treated cells. (A) The level of H3K18la in LPS and lactic acid-treated cells compared with the control group. (B) The enrichment of H4K5la and H3K18la in the promoter regions of LINC00152 had no significant difference between the LPS treated group and the control group. [file Image_2.tif]
